# Air versus long-acting gas tamponade for idiopathic macular hole surgery with 24-hour face-down positioning: a bicentric retrospective study

**DOI:** 10.1186/s40942-026-00908-0

**Published:** 2026-08-11

**Authors:** Benjamin Jacob, Eric Parrat, Umberto Lorenzi

**Affiliations:** 1 Department of Ophthalmology, Rouen University Hospital, Rouen, France; 2 Clinique Les Eaux Claires, Baie-Mahault, Guadeloupe France

**Keywords:** Macular hole, Vitrectomy, Air tamponade, Gas tamponade, Internal limiting membrane, Face-down positioning

## Abstract

We compared air and gas tamponade for idiopathic full-thickness macular hole (FTMH) surgery with a 24-hour face-down protocol, in a bicentric, retrospective real-world series of 36 air- and 19 gas-treated eyes. Allocation was non-random (gas predominated earlier, air later). Preoperative symptom duration was short in both groups (median 1 [1–2] months); median hole diameter was 456 [312–611] µm (air) and 497 [364–776] µm (gas; *p* = 0.40). FTMH closure at 1 month was 100% (36/36) with air and 94.7% (18/19) with gas (*p* = 0.35), reaching 100% in both groups at 3 and 6 months. Best-corrected visual acuity improved significantly within each group, with no significant between-group difference at any timepoint. No complications occurred with air, whereas peripheral retinal breaks occurred only in the gas group. The air group was younger than the gas group (66.1 vs. 74.4 years; *p* = 0.018). Air was associated with anatomical and functional outcomes comparable to gas in this cohort; however, the non-random allocation, small sample, absence of a pre-specified margin and limited statistical power preclude any formal non-inferiority conclusion. Air with short positioning appears a practical alternative to gas in sub-acute holes across the size spectrum, warranting prospective confirmation.

Long-acting gas remains the reference tamponade for idiopathic full-thickness macular hole (FTMH) surgery, but it imposes prolonged visual impairment, positioning constraints and an air-travel restriction until resorption [1]. Air is an attractive short-acting alternative, resorbing within about ten days [2], avoiding altitude restriction and the high global-warming potential of fluorinated gases, with reported closure rates of 75–100% [3–6]. Whether air performs as well as gas—particularly for large holes and with abbreviated positioning [7, 8]—remains debated. We therefore compared air and gas tamponade, each with a 24-hour face-down protocol, in a real-world bicentric cohort.

## Methods

This retrospective study (Rouen University Hospital, France; Clinique Les Eaux Claires, Guadeloupe; September 2020–June 2024) included consecutive eyes undergoing pars plana vitrectomy with dye-assisted internal limiting membrane (ILM) peeling for idiopathic FTMH, treated with air (*n* = 36) or gas (*n* = 19; C2F6 = 15, SF6 = 3, C3F8 = 1, pooled as long-acting gases). Allocation was not randomised: gas predominated earlier in the study period and air progressively replaced it as practice evolved. Secondary holes (traumatic, myopic > − 6 D, retinal-detachment-associated) and prior vitreoretinal surgery were excluded. Data were collected using Dateye (dateye.ai). Holes were classified by minimum width per the International Vitreomacular Traction Study (IVTS) group [9]. All patients maintained 24-hour face-down positioning; overnight admission was used solely to verify posture compliance and is not intrinsic to the protocol. The primary outcome was optical coherence tomography (OCT)-confirmed closure at 1 month; secondary outcomes were closure at 3 and 6 months, best-corrected visual acuity (BCVA, Early Treatment Diabetic Retinopathy Study [ETDRS] letters) and safety. Skewed continuous variables (Shapiro–Wilk) are reported as median [interquartile range, IQR]; between-group comparisons used the Mann–Whitney U test (continuous) and Fisher’s exact test (categorical), and within-group BCVA change the Wilcoxon signed-rank test (Python/SciPy); *p* < 0.05 was significant. No non-inferiority margin was pre-specified, and the study was not powered for a formal non-inferiority conclusion; comparisons are exploratory. The study followed the Declaration of Helsinki, with oral informed consent for data reuse obtained at the preoperative consultation.

## Results

Baseline characteristics are shown in Table 1. Groups were comparable except for age, the air group being younger than the gas group (66.1 ± 11.6 vs. 74.4 ± 8.4 years; *p* = 0.018). Symptom duration was short in both groups (median 1 [1–2] months, maximum 3), indicating sub-acute holes, and hole diameter did not differ significantly (456 [312–611] vs. 497 [364–776] µm; *p* = 0.40). Flap-based ILM techniques predominated in both groups (35/36, 97.2% with air; 18/19, 94.7% with gas), the inverted flap being most frequent (41.7% and 73.7%, respectively). The peeling technique was left to the surgeon’s discretion, the most appropriate flap being chosen intraoperatively according to the situation; the full distribution by group and by hole size is given in Table 2.

**Table 1:** Baseline characteristics by tamponade group

Characteristic	Air (*n* = 36)	Gas (*n* = 19)	*p*
Age, years (mean ± SD)	66.1 ± 11.6	74.4 ± 8.4	0.018
Female sex	26 (72.2%)	10 (52.6%)	0.233
Left eye	22 (61.1%)	6 (31.6%)	0.050
Symptom duration, months (median [IQR])	1 [1–2]	1 [1–2]	0.492
Hole diameter, µm (median [IQR])	456 [312–611]	497 [364–776]	0.400
Baseline BCVA, ETDRS (mean ± SD)	51.2 ± 12.8	44.6 ± 12.4	0.148
Axial length, mm (mean ± SD)	24.6 ± 3.3	24.5 ± 2.4	0.594
Vitreomacular traction	8 (22.2%)	3 (15.8%)	0.730
Pseudophakic	14 (38.9%)	9 (47.4%)	0.577
Combined phacovitrectomy	9 (25.0%)	3 (15.8%)	0.511

Continuous variables as mean ± SD or median [IQR]; categorical as n (%). Between-group comparisons: Mann–Whitney U or Fisher’s exact test. The gas group pools C2F6, SF6 and C3F8

**Table 2:** Distribution of ILM peeling techniques by group

ILM peeling technique	Air (*n* = 36)	Gas (*n* = 19)
Inverted flap	15 (41.7%)	14 (73.7%)
Temporal flap + peri-hole ILM removal	11 (30.6%)	1 (5.3%)
Superior flap + peri-hole ILM removal	8 (22.2%)	0 (0%)
Free flap	0 (0%)	2 (10.5%)
Temporal flap, nasal ILM preserved	1 (2.8%)	1 (5.3%)
Complete ILM peeling	1 (2.8%)	1 (5.3%)

Values are n (%). In the air group, technique use by hole size was: large (> 400 μm, *n* = 22) — inverted 8, temporal 7, superior 7; medium (250–400 μm, *n* = 11) — inverted 6, temporal 4, superior 1; small (< 250 μm, *n* = 3) — inverted 1, complete peeling 1, nasal-sparing temporal 1. ILM = internal limiting membrane

**Fig. 1:**
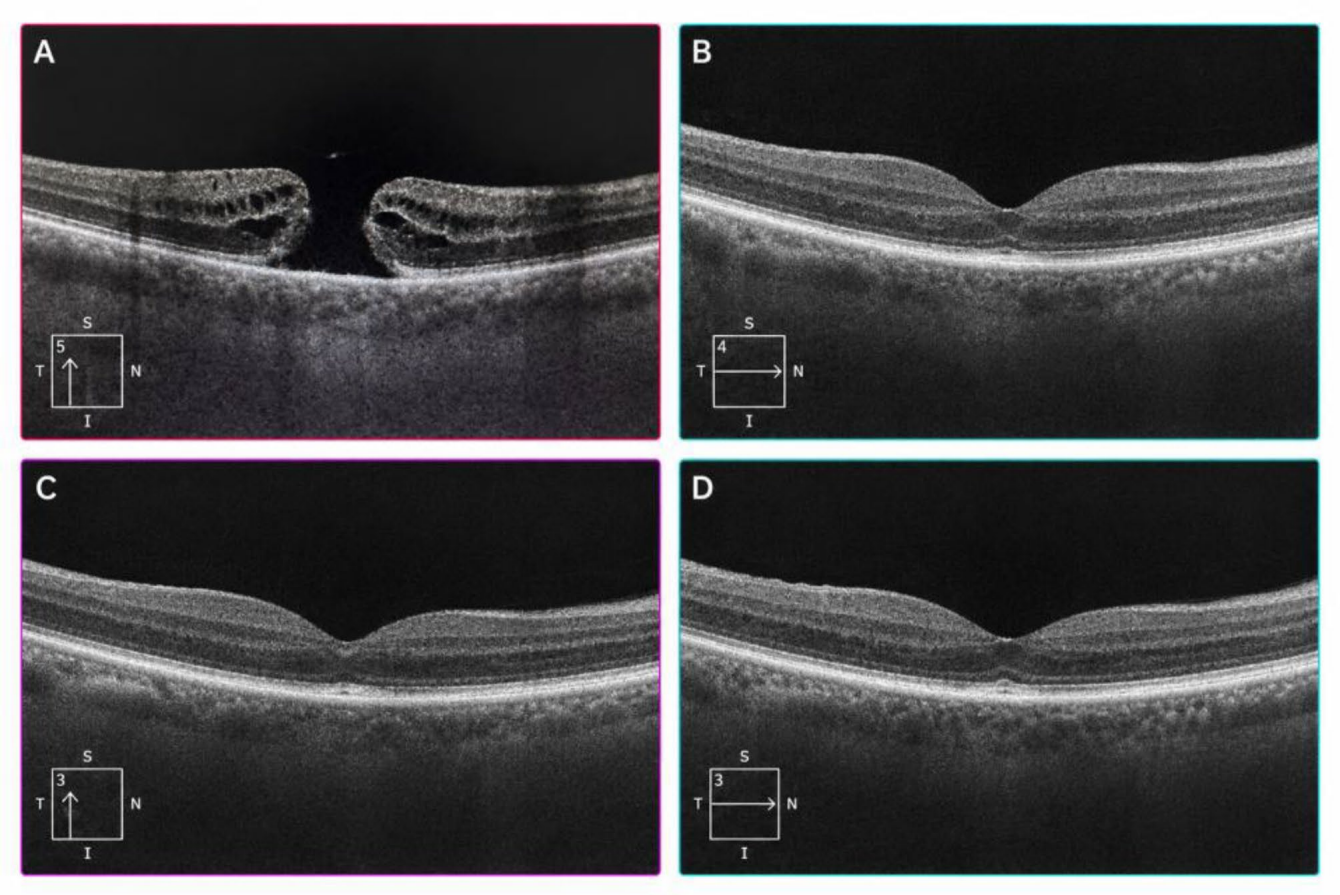
Representative spectral-domain OCT of an idiopathic FTMH treated with vitrectomy, ILM peeling and air tamponade: (**A**) preoperatively and (**B**) 1 month, (**C**) 3 months and (**D**) 6 months postoperatively, showing complete and durable closure with progressive restoration of the foveal contour

FTMH closure at 1 month was 100% (36/36) with air and 94.7% (18/19) with gas (*p* = 0.35), reaching 100% in both groups at 3 and 6 months; a representative case is shown in Fig. 1. For air, closure was complete within every IVTS size stratum, including all 22 large holes (> 400 μm). The decreasing numbers of eyes at 3 and 6 months reflect routine loss to clinical follow-up rather than anatomical failure or reopening; no reopening was observed.

**Fig. 2:**
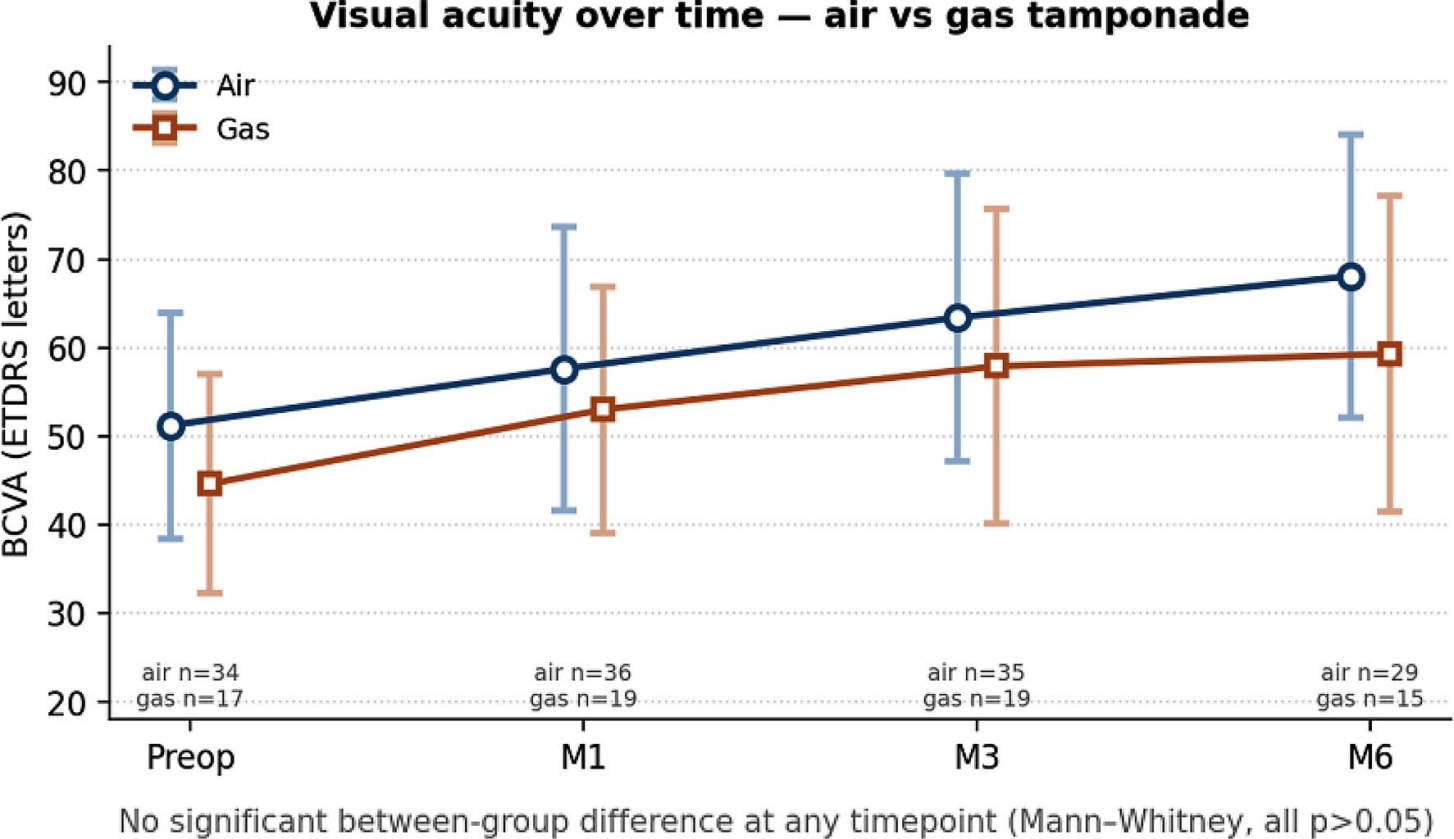
Visual acuity (ETDRS letters, mean ± SD) over follow-up in the air and gas groups. No significant between-group difference at any timepoint (Mann–Whitney U test). The number of eyes assessed at each visit is shown beneath the axis for each group

BCVA improved significantly from baseline within each group (mean within-eye paired gain at 6 months: air + 18.6 letters, *p* < 0.0001; gas + 13.8 letters, *p* = 0.015; Wilcoxon signed-rank), with no significant between-group difference at any timepoint (Table 3; Fig. 2). Confidence intervals were wide, consistent with the modest sample size.

**Table 3:** Best-corrected visual acuity (ETDRS letters) over time by group

Timepoint	Air (mean ± SD)	Gas (mean ± SD)	*p*
Preoperative	51.2 ± 12.8	44.6 ± 12.4	0.148
1 month	57.6 ± 16.0	53.0 ± 13.9	0.220
3 months	63.4 ± 16.2	57.9 ± 17.8	0.150
6 months	68.1 ± 16.0	59.3 ± 17.8	0.069

Between-group comparisons: Mann–Whitney U test

## Safety

No intraoperative or postoperative complications occurred in the air group, whereas peripheral retinal breaks occurred in 3 gas-treated eyes (15.8%). These breaks were absent on preoperative examination and were detected intraoperatively during induction of posterior vitreous detachment; all were treated with laser retinopexy or cryotherapy.

## Discussion

In this real-world bicentric series, air tamponade with 24-hour face-down positioning was associated with anatomical and functional outcomes comparable to long-acting gas, with a favourable safety profile and reduced postoperative burden. In this series, closure in the air group was complete across the full size spectrum, including large holes, and visual gains did not differ between groups. These observations suggest that air yields outcomes comparable to gas.

The uniformly high closure most plausibly reflects the near-systematic use of flap-assisted ILM techniques (inverted flap [10]), which promote closure largely independently of the tamponade. Our data therefore suggest that it is the combination of a flap-assisted technique with air tamponade and short face-down positioning—rather than the tamponade alone—that underlies these favourable outcomes; this hypothesis is consistent with the recognised impact of flap techniques on closure and merits dedicated study. Closure of every large hole, up to 923 μm, is in line with reports that flap techniques extend high closure rates to large and very large (> 600 μm) holes [10]. The absence of prolonged positioning is likewise supported by randomised evidence that face-down posturing may be unnecessary even for large holes [8], and by series reporting equivalent tamponade with room air compared with SF6 [4].

Several limitations temper these findings. The study was retrospective and non-randomised: gas predominated earlier and air later, so a learning-curve effect is potentially major—over the study period the surgeons’ experience, the surgical indications and the flap techniques all evolved, and part of the apparent performance of air may reflect improving surgery rather than the tamponade. The air group was also significantly younger (66 vs. 74 years); because younger patients often recover vision better, this imbalance may have favoured air and could mask part of the functional comparison. The gas subtypes were pooled despite differing durations, follow-up was incomplete, and closure showed a ceiling effect. As is inherent to this retrospective design, we do not have an exact count of all eyes screened but excluded over the period, which we acknowledge as a further limitation. Critically, the absence of a between-group difference is not evidence of equivalence: with 36 versus 19 eyes and near-complete closure, the study lacked the power to exclude a clinically relevant difference, no non-inferiority margin was pre-specified, and the cohort was entirely sub-acute (symptom duration ≤ 3 months), precluding extrapolation to chronic holes.

We deliberately did not perform multivariable adjustment: with a small sample and a single non-closure in the whole cohort, a logistic model would not converge, and adjusting the visual outcome for several covariates would risk overfitting; the comparisons are therefore unadjusted and exploratory. Overnight admission to verify posturing may also be costly or impractical in some settings and limits external validity, although the intervention itself requires only 24 h of positioning. These results are hypothesis-generating and warrant a prospective, adequately powered non-inferiority trial with a pre-specified margin, randomising tamponade and positioning duration.

## Conclusion

Air tamponade with 24-hour face-down positioning was associated with anatomical and functional outcomes comparable to gas and a favourable safety profile in this non-randomised real-world series. The data suggest outcomes comparable to gas but do not permit a formal non-inferiority conclusion; they support air with short positioning as a practical alternative worthy of prospective confirmation.

## Data Availability

The datasets generated and analysed during the current study are available from the corresponding author on reasonable request.

## Declarations

### Use of generative AI and AI-assisted technologies

During the preparation of this work the authors used Claude (Anthropic) to assist with statistical analysis, drafting and language refinement. The authors reviewed and edited all content and take full responsibility for it.

## Abbreviations

BCVA: Best-corrected visual acuity
ETDRS: Early Treatment Diabetic Retinopathy Study
FTMH: Full-thickness macular hole
ILM: Internal limiting membrane
IQR: Interquartile range
IVTS: International Vitreomacular Traction Study
OCT: Optical coherence tomography
SD: Standard deviation
